# Bilateral Parkinson’s disease model rats exhibit hyperalgesia to subcutaneous formalin administration into the vibrissa pad

**DOI:** 10.1371/journal.pone.0225928

**Published:** 2019-12-05

**Authors:** Hiroharu Maegawa, Nayuka Adachi, Hiroshi Hanamoto, Chiho Kudo, Hitoshi Niwa

**Affiliations:** Department of Dental Anesthesiology, Osaka University Graduate School of Dentistry, Suita, Osaka, Japan; Florey Institute of Neuroscience and Mental Health, The University of Melbourne, AUSTRALIA

## Abstract

We bilaterally injected 6-hydroxydopamine (6-OHDA) into the medial forebrain bundle of rats and developed bilateral Parkinson’s disease (PD) model rats in order to experimentally investigate the neural mechanisms underlying the alteration of nociception in the orofacial region of patients with PD. We explored the effects of dopamine depletion on nociception by investigating behavioral responses (face rubbing) triggered by subcutaneous administration of formalin into the vibrissa pad. We also assessed the number of c-Fos–immunoreactive (c-Fos-IR) cells in the superficial layers of the trigeminal spinal subnucleus caudalis (Vc). Subcutaneous formalin administration evoked a two-phase increase in face rubbing. We observed the first increase 0–5 min after formalin administration (first phase) and the second increase 10–60 min after administration (second phase). The number of face rubbing behaviors of 6OHDA–injected rats did not significantly change compared with saline–injected rats in both phases. Significant increase of c-Fos-IR cells in the Vc was found in 6-OHDA–injected rats after formalin administration compared with those in saline–injected rats after formalin administration. We also assessed expression of c-Fos-IR cells in the paraventricular nucleus (PVN), and significant decrease of c-Fos-IR cells in the PVN of 6-OHDA–injected rats was found. Taken together, these findings suggest that bilateral dopaminergic denervation evoked by 6-OHDA administration causes hyperalgesia in the trigeminal region and the PVN may be involved in the hyperalgesia.

## Introduction

Reportedly, 40%–85% of patients with Parkinson’s disease (PD) experience pain [1]. Previous study has suggested that the dopaminergic system within the basal ganglia plays key roles in handling noxious information [2]. Previously, we reported that unilateral PD model rats showed a significant gain in face rubbing and expression levels of c-Fos in the trigeminal spinal subnucleus caudalis (Vc) following subcutaneous administration of formalin into the vibrissa pad [3]. We proposed that unilateral dopamine reduction in the nigrostriatal system evokes hyperalgesia by nociceptive stimulation in the trigeminal region. Several studies have reported responses to noxious stimuli in unilateral PD model rats [4–6]; however, the mechanisms underlying the alteration of nociception in PD model rats remain unclear. These studies, including ours, involved the use of unilateral PD model rats. In few studies, nociception has been examined with the usage of bilateral PD model rats [7–9]. Because idiopathic PD is bilateral, bilateral PD model rats are more closely to the pathological condition in humans [10].

The aim of this research was to explore changes in the neural mechanisms of nociception in the trigeminal region in patients with PD. Therefore, we developed bilateral PD model rats by 6-hydroxydopamine (6-OHDA) administration into the medial forebrain bundle (MFB) [11]; we then examined behavioral and immunohistochemical reactions to subcutaneous administration of formalin into the vibrissa pad of the rats.

## Material and methods

All the protocols were performed in accordance with the ethical guidelines of the International Association for the Study of Pain [12], and were approved by the Osaka University Graduate School of Dentistry Animal Care and Use Committee (26-015-0). We produced unilateral PD model rats in our previous study [3], and no rats died after unilateral 6-OHDA injection. Since we have never produced bilateral PD model rats, the mortality rate of bilateral 6-OHDA–injected rats was not understood well at the planning stage of the study and the beginning of the study. However, in the plan document of the present study, we wrote that the mortality rate of bilaterally 6-OHDA–injected rats may be high compared with that of unilaterally 6-OHDA–injected rats. And, our Animal Care and Use Committee reviewed and approved the mortality aspects of the protocol.

### 6-OHDA injection

Male Wistar rats (170–200g, SLC, Hamamatsu, Japan) were used. Rats were maintained in a 12 h dark/light cycle, and can take food and water freely. The procedure of 6-OHDA injection was performed as reported previously [3]. Although 6-OHDA injection was unilateral in our previous report, injections were bilateral in the present study. Briefly, 30 minutes before the surgical procedure, rats received an administration of desipramine hydrochloride (20 mg/kg, Sigma, St. Louis, MO, USA) intraperitoneally to avoid destruction of the noradrenergic neurons. Then, the rats were anesthetized intraperitoneally by administration of a saline solution with midazolam (2.0 mg/kg, Sandoz, Tokyo, Japan), medetomidine (0.375 mg/kg, Zenoaq, Fukushima, Japan), and butorphanol (2.5 mg/kg, Meiji Seika Pharma, Tokyo, Japan). Rats were fixed in position with a stereotaxic apparatus (Narishige, Tokyo, Japan) which incisor bar was placed 2.4 mm below the interaural plane [3]. Next, 1.5 mg of 6-OHDA (Sigma, St. Louis, MO, USA), which was dissolved in 0.5 ml of sterile saline that contained 0.01% ascorbic acid, was bilaterally injected into the MFB. The injection sites of 6-OHDA were as follows: anteroposterior 2.2 mm caudal from the bregma, mediolateral ± 1.4 mm from the midline, dorsoventral 6.5 mm from dural surface (2 μl injection). The administration of 6-OHDA was performed through a cannula connected to a microinjection pump (KD scientific, Holliston, MA, USA) at 60 μl/h and it was kept in place for 5 min after the end of administration. For control rats, saline was similarly administered. Postoperatively, 6-OHDA–injected rats were fed (DietGel 76A, ClearH_2_O, Portland, ME, USA) with a tube for oral administration for rodents (FTP-18-38, Primetech Corporation, Tokyo, Japan). They were fed 3 times per day during 10–13 days after surgery. We measured body weight of the rats and observed their actions once a day. We set humane endpoint. We planned to perform euthanasia when rats show the decrease of body weight more than 20% compared with control rats or severe diarrhea or severe vomiting or persisting crouching position. No rats fulfilled the criteria. Since we used 10 rats in each group in our previous study [3], we planned to use 10 rats in each group in the present study. Although we injected 6-OHDA into the MFB bilaterally for 68 rats, which is similar number of rats used in our previous study, we could produce only 10 bilateral PD model rats. And, 54 rats showed unilateral dopaminergic denervation, and 4 rats died. The mortality rate of 6-OHDA–injected rats is 5.9% [ = 4 (dead rats) / 68 (6-OHDA–injected rats)]. And the mortality rate of bilateral PD model rats is 28.6% [ = 4 (dead rats) / 4 (dead rats) + 10 (bilateral PD model rats)]. It was difficult to accurately inject 6-OHDA bilaterally into the MFB. The mortality rate of bilateral 6-OHDA–injected rats was reported over 60% [13]. Because no rats died after surgery in our previous study [3], we thought that the mortality rate of 6-OHDA–injected rats in the present study is high. Since the number of rats that we cannot use subsequent experiments (dead rats or unilateral PD model rats) may increase, we thought that attempting to produce more bilateral PD model rats may be difficult in the context of ethical perspective. The number of saline–injected rats was matched with the number of the 6-OHDA–injected rats.

### Formalin test

Two weeks after 6-OHDA or saline administration into the MFB, we subcutaneously administered formalin to rats. Before the test, we put the rats in a clear Plexiglas box and allowed them to acclimatize to the circumstances for at least 60 min. There were 2 experimental groups: (i) rats bilaterally injected with 6-OHDA into the MFB (6-OHDA) and (ii) rats bilaterally injected with saline into the MFB (sham). Subcutaneous administration of 4% formalin (50 μl) was performed into the left vibrissa pad of rats. Then, we returned the rats to the box and observed their face rubbing behavior. The number of face rubbing behaviors was counted every 5 min for 60 min. The experimenter engaged in observation of behavior was blinded to the procedures performed on the rats.

### Immunohistochemistry

Two hours after subcutaneous formalin administration, the rats were intraperitoneally administered an overdose of sodium pentobarbital, and perfusion was done intracardially with 150 ml of phosphate-buffered saline (PBS, 0.02 M) and then with 350 ml of 4% paraformaldehyde dissolved in phosphate buffer (PB, 0.1 M). We removed the brains and immersed them in the same solution overnight for post-fixing, and then soaked them in 30% sucrose solution made by PB. Perfusion was also conducted for rats without subcutaneous administration of formalin 2 weeks after 6-OHDA or saline administration into the MFB.

To detect destruction of dopaminergic neurons, immunohistochemistry for tyrosine hydroxylase (TH) was conducted in all the rats. A freezing microtome was used to make serial coronal sections of the striatum and substantia nigra (SN) at a 60 μm thickness. Immersion of the sections to methanol containing 0.3% hydrogen peroxide was done for 20 min, incubation of them in 1% normal horse serum (Vector, Burlingame, CA, USA) was performed for 30 min. Then, the sections were incubated with mouse monoclonal anti-TH antibody (1:8000 dilution, Sigma, T2928, antigen: rat TH, St. Louis, MO, USA, RRID: AB_477569) for 12 h. Incubation of the sections with biotinylated horse anti-mouse antibody (1:200 dilution, Vector, BA-2000, Burlingame, CA, USA, RRID: AB_2313581) and incubation of them with avidin-biotin-peroxidase complex (Vectastain ABC Elite Kit, Vector, Burlingame, CA, USA) was performed for 1 h respectively. The sections were visualized using diaminobenzidine tetrahydrochloride (DAB) (DAB substrate kit, Vector, Burlingame, CA, USA). Then, the sections were mounted on gelatin-coated glass slides, and air dried at room temperature. Counterstaining was done with cresyl violet before mounting under a coverslip. For every third section, we examined TH–immunoreactive (TH-IR) cells and cells stained with cresyl violet (TH-negative cells) in the SN pars compacta (pc) bilaterally using light microscopy. The number of TH-IR cells and TH-negative cells were counted every third sections (180 μm apart), and total number of TH-IR cells and TH-negative cells (/10 sections / rat) were calculated.

To explore the activity of the Vc and paraventricular nucleus (PVN) neurons, immunohistochemistry for c-Fos was performed. A freezing microtome was used to produce serial coronal sections of the Vc and PVN at a 60 μm thickness. Sections were immersed in 1% normal goat serum (Vector, Burlingame, CA, USA) for 30 min and incubated with monoclonal rabbit anti-c-Fos antibody (1:7000 dilution, Cell Signaling Technology, 2250, antigen: a synthetic peptide corresponding to the sequence of human c-Fos, Danvers, MA, USA, RRID: AB_2247211) for 12 h, followed by immersion with biotinylated goat anti-rabbit antibody (1:200 dilution, Vector, BA-1000, Burlingame, CA, USA, RRID: AB_2313606) for 1 h. Then, subsequent reactions and visualizations were performed as mentioned previously. For every third section, we examined c-Fos–immmunoreactive (c-Fos-IR) cells in the Vc using light microscopy. A c-Fos-IR cell count in the superficial layers of the Vc on the side ipsilateral to the side where formalin was injected was performed for sections containing the obex to the section 5 mm caudal to the obex. Five sections containing the most stained cells were selected from each rat, and then, calculation of the mean number of c-Fos-IR cells in the Vc/section was performed. We also counted c-Fos-IR cells in the PVN for every third section, and calculated the total number of cells. The experimenter engaged in count of c-Fos-IR cells was blinded to the procedures performed on the rats.

### Statistical analysis

The results are presented as mean ± SEM. Comparison of behavioral data was performed using an unpaired *t*-test, and comparison of immunohistochemical data was done using two-way analysis of variance (ANOVA) followed by Bonferroni’s test for multiple comparison (IBM, SPSS Statistics ver. 24). Differences were considered statistically significant at *p* < 0.05.

## Results

### Immunohistochemical labeling for TH

**Fig 1:**
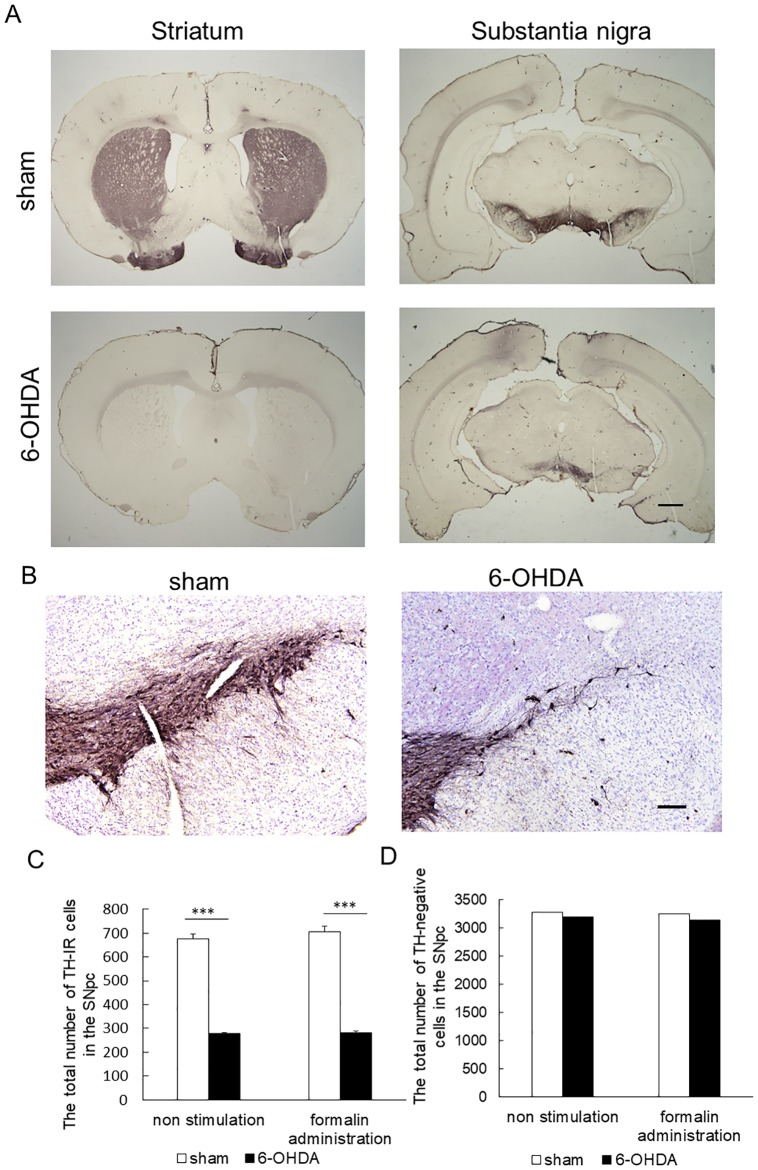
Photomicrographs of immunohistochemical labeling for tyrosine hydroxylase (TH) and histological analysis. (A) Photomicrographs of immunohistochemical labeling for TH of the striatum and substantia nigra (SN). TH immunoreactivity was visualized in sections using diaminobenzidine tetrahydrochloride (DAB). Immunoreactivity for TH was high in the bilateral striatum and SN in sham rats with bilateral saline administration into the medial forebrain bundle (MFB). Rats with bilateral 6-OHDA administration into the MFB indicated a striking reduction in TH immunoreactivity in the striatum and SN on both sides. Scale bar stands for 1mm. (B) Photomicrographs of the SN pars compacta (pc) cells. TH–immunoreactive (TH-IR) cells visualized with diaminobenzidine tetrahydrochloride (DAB) and cells stained with cresyl violet (TH-negative cells) in the SNpc were found in sham rats and 6-OHDA rats. Scale bar stands for 100 μm. (C) The total number of TH-IR cells in the SNpc (mean ± SEM, ***p < 0.001). The number of TH-IR cells in 6-OHDA rats without formalin administration was significantly lower than that of sham rats. The number of TH-IR cells in 6-OHDA rats with formalin administration was significantly lower than that of sham rats. (D) The total number of TH-negative cells in the SNpc (mean ± SEM). No significant difference was found between the number of TH-negative cells of 6-OHDA rats and that of sham rats with and without formalin administration.

We performed immunochistochemical detection of TH in all the rats. Immunoreactivity of TH was high in the bilateral striatum and SN of sham rats (formalin injection: n = 5, non-stimulation: n = 5) that received bilateral saline injections into the MFB (Fig 1A, S1 and S3 Figs). Conversely, 6-OHDA rats (formalin injection: n = 5, non-stimulation: n = 5) that received bilateral 6-OHDA administrations displayed distinct reduction in TH immunoreactivity in the striatum and SN on both sides (Fig 1A, S2 and S4 Figs). TH-IR-cells visualized with diaminobenzidine tetrahydrochloride (DAB) and TH-negative cells stained with cresyl violet in the SNpc were found in sham rats and 6-OHDA rats (Fig 1B, S5 and S6 Figs). The number of TH-IR cells of 6-OHDA rats without formalin administration was significantly lower than that of sham rats (278.4 ± 2.8 vs. 675.2 ± 20.0, *p* < 0.001, Fig 1C, S1 File). It showed a 58.8% TH-IR cell loss in the SNpc of 6-OHDA rats compared with sham rats. The number of TH-IR cells of 6-OHDA rats with formalin administration was significantly lower than that of sham rats (283.2 ± 5.2 vs. 707.4 ± 22.6, *p* < 0.001, Fig 1C, S1 File). It showed a 60.0% TH-IR cell loss in the SNpc of 6-OHDA rats compared with sham rats. No significant difference was found between the number of TH-negative cells in 6-OHDA rats and in sham rats (3273.8 ± 39.0 vs. 3190.4 ± 72.0 without formalin administration, 3241.6 ± 91.6 vs. 3138.4 ± 42.6 with formalin administration, Fig 1D, S1 File).

### Formalin test

**Fig 2:**
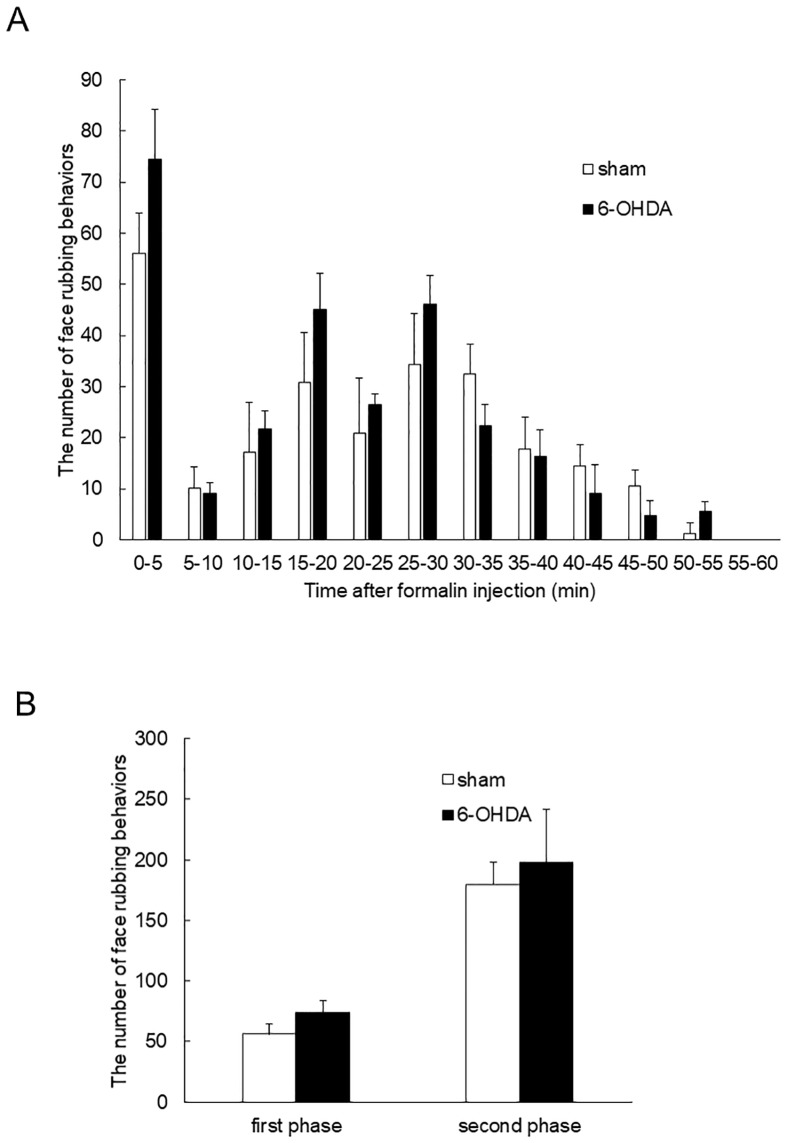
Number of face rubbing behaviors induced by subcutaneous formalin injection into the vibrissa pad. (A) The number of face rubbing behaviors per 5 min (mean ± SEM) of both groups. Subcutaneous formalin administration induced a two-phase increase in face rubbing behavior. (B) The number of face rubbing behaviors in the first phase (0–5 min) and second phase (10–60 min) (mean ± SEM). The number of face rubbing behaviors of rats with bilateral 6-OHDA administration did not change significantly compared with that of rats with bilateral saline administration in both phases (Fig. 2B).

Face rubbing behaviors were observed following subcutaneous administration of formalin into the vibrissa pad. We observed a two-phase increase in face rubbing behavior after subcutaneous formalin administration (Fig 2A, S1 File). Immediately after formalin administration, the first increase in face rubbing behavior began and continued for about 5 min; this reaction was defined as the first phase. Following this, we observed a second increase in face rubbing behavior, which lasted from 10 to 60 min after the injection; this was defined as the second phase. We calculated the mean number of face rubbing behaviors in each phase. The number of face rubbing behaviors of 6-OHDA rats did not change significantly compared with that of sham rats in the first phase (74.4 ± 9.7 vs. 56.0 ± 8.0, Fig 2B, S1 File). In the second phase, the number of face rubbing behaviors of 6-OHDA rats did not change significantly compared with that of sham rats (198.0 ± 18.2 vs. 179.6 ± 43.7, Fig 2B, S1 File).

### Immunohistochemical labeling for c-Fos

**Fig 3:**
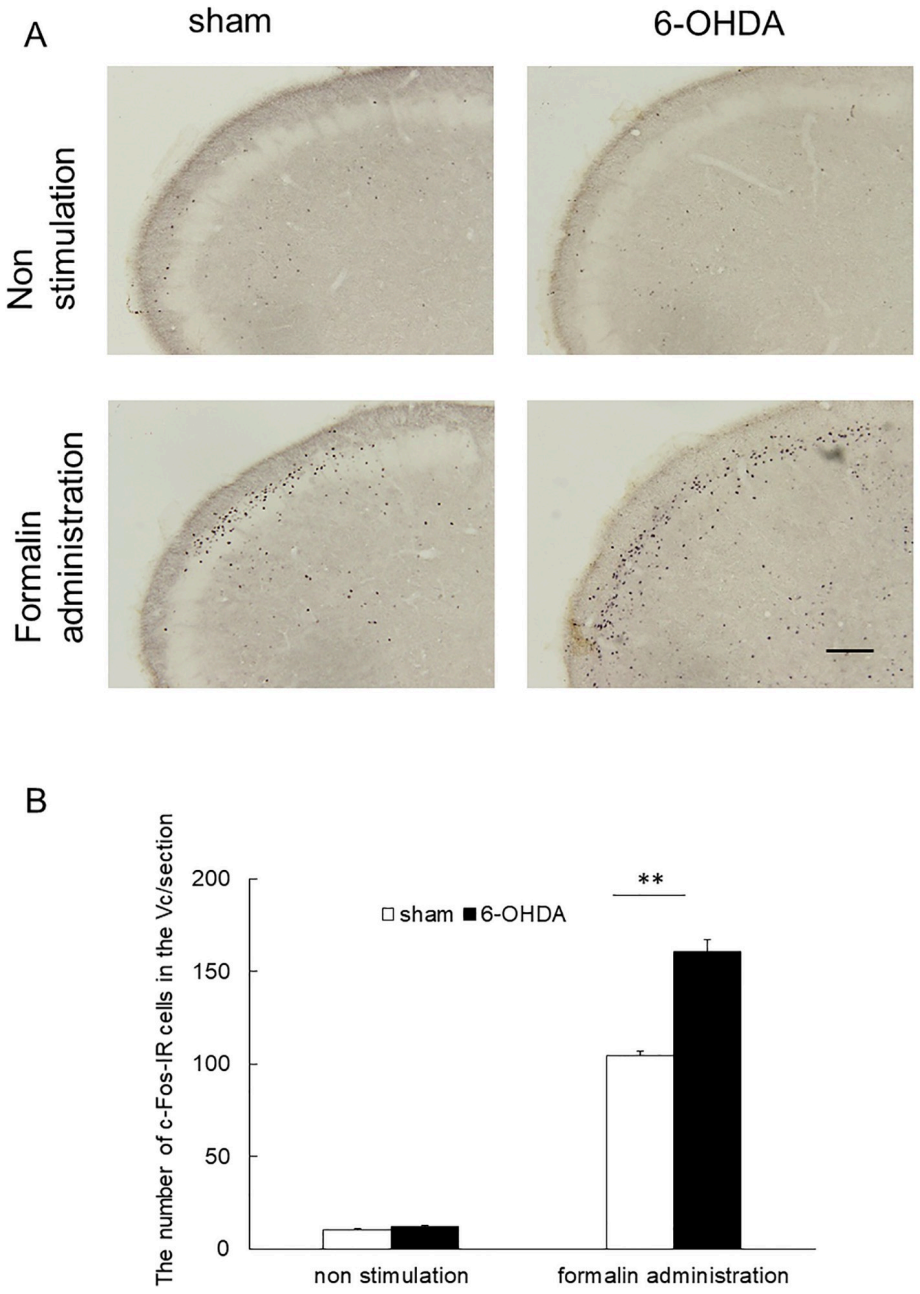
Immunohistochemical labeling for c-Fos in the trigeminal spinal subnucleus caudalis (Vc) induced by subcutaneous formalin administration into the vibrissa pad. (A) Photomicrographs of c-Fos–immunoreactive (c-Fos-IR) cells in the Vc. Immunoreactivity of c-Fos was visualized in sections using DAB. In rats with bilateral 6-OHDA or saline administration that received subcutaneous formalin administration, c-Fos-IR cells were found mostly in the superficial layers of the Vc. Scale bar stands for 200 μm. (B) Significantly increased number of c-Fos-IR cells were found in 6-OHDA rats with subcutaneous formalin administration compared with sham rats with formalin administration (mean ± SEM, **p < 0.01). In the groups without formalin administration, 6-OHDA rats showed no significant change in the number of c-Fos-IR cells compared with sham rats.

In the groups that received subcutaneous formalin administration, c-Fos-IR cells were found mostly in the superficial layers of the Vc, and the largest number was found 1.4 mm caudal to the obex (Fig 3A, S9 and S10 Figs). In the groups that received no stimulation, few c-Fos-IR cells were found rostrocaudally over the entire extent of the Vc. Therefore, we selected sections that were around 1.4 mm caudal to the obex as with the groups that received subcutaneous formalin administration (S7 and S8 Figs). A significant increase of c-Fos-IR cells was found in 6-OHDA rats with subcutaneous formalin administration compared with that in sham rats (160.5 ± 6.5 vs. 104.5 ± 2.1, *p* < 0.01, Fig 3B, S1 File). In the groups without formalin administration, 6-OHDA rats showed no significant change in c-Fos-IR cells compared with saline rats (12.2 ± 0.6 vs. 10.4 ± 0.5, Fig 3B, S1 File).

**Fig 4:**
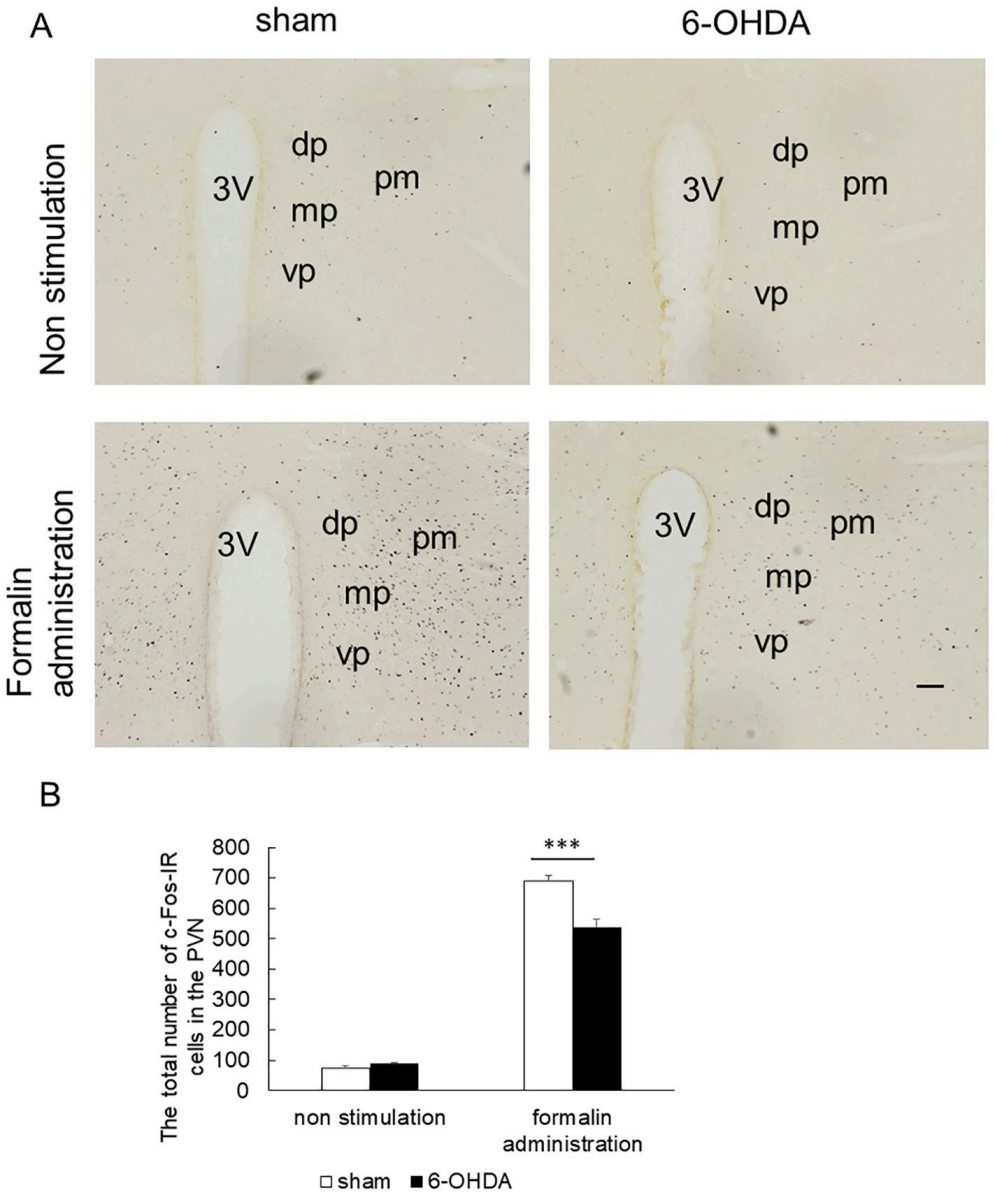
Immunohistochemical labeling for c-Fos in the PVN. (A) Photomicrographs of c-Fos–immunoreactive (c-Fos-IR) cells in the PVN. DAB was used for visualizing c-Fos immunoreactivity. 3V: third ventricle, dp: dorsal parvicellular, mp: medial parvicellular, vp: ventral parvicellular, pm: posterior magnocellular. Scale bar stands for 100 μm. (B) The total number of c-Fos-IR cells in the PVN (mean ± SEM, ***p < 0.001). No significant difference was found in the total number of c-Fos-IR cells in the PVN between 6-OHDA rats without formalin administration and sham rats without formalin administration. The total number of c-Fos-IR cells in the PVN of 6-OHDA rats with formalin administration was significantly lower than that of sham rats with formalin administration.

We found c-Fos-IR cells in the entire extent of the PVN rostrocaudally (Fig 4A, S11–S14 Figs); hence, we counted the cells bilaterally and calculated the total number of the cells. No significant difference was found in the total number of c-Fos-IR cells in the PVN between 6-OHDA rats without formalin administration and sham rats without formalin administration (88.8 ± 3.4 vs. 75.8 ± 4.2, Fig 4B, S1 File). The total number of c-Fos-IR cells in the PVN of 6-OHDA rats with formalin administration was significantly lower than that of sham rats with formalin administration (536.6 ± 26.1 vs. 689.8 ± 17.5, *p* < 0.001, Fig 4B, S1 File).

## Discussion

The principal finding of this study was the fact that bilateral nigrostriatal lesions induced by 6-OHDA administration into the MFB increased c-Fos expression in the Vc following subcutaneous formalin administration into the vibrissa pad. This finding suggests that hyperalgesia occur in the orofacial region of bilateral PD model rats.

Formalin injection was reported to elicit a two-phase response [14]. Previously, we reported that unilateral PD model rats displayed increased face rubbing behaviors and increased the c-Fos-IR cell number in the Vc following formalin injection into the vibrissa pad, suggestive of hyperalgesia in the orofacial region [3]. Conversely, the number of face rubbing behaviors of 6-OHDA rats did not change significantly compared with sham rats in this study, although 6-OHDA rats showed an increasing trend in the number of face rubbing behaviors compared with sham rats. Therefore, this result does not suggest alteration of nociception in rats bilaterally administered with 6-OHDA. However, it was reported that PD model rats that received bilateral 6-OHDA injections showed movement disorders [15]. The number of face rubbing behaviors in this study may have been affected by the movement disorder induced by bilateral 6-OHDA injection. Therefore, evaluating changes in nociception using only behavioral responses appears to be limited for bilateral 6-OHDA injected rats.

Next, we performed immunohistochemical detection of c-Fos to investigate changes in nociception in bilateral 6-OHDA injected rats. The expression levels of c-Fos reflect activities of the dorsal horn neurons in the first and second phases of the formalin test [16]. Therefore, we utilized c-Fos as an index of the action of the Vc neurons in the orofacial formalin testing period. In the present study, an increase in c-Fos-IR cell number in the Vc was found in 6-OHDA rats with formalin administration compared with sham rats with formalin administration. This finding suggests hyperalgesia occurs in rats that were bilaterally injected with 6-OHDA.

Previous studies using unilateral PD model rats reported alterations of nociception [3–6]. In the present study, we found that bilateral 6-OHDA injected rats showed an increase in c-Fos-IR cell numbers in the Vc following subcutaneous formalin administration into the vibrissa pad. This finding suggests that dopaminergic denervation elicits hyperalgesia, which is consistent with previous studies reporting changes in nociception. However, the precise mechanism underlying this response remains unclear.

In the present study, we found a decrease in the c-Fos-IR cell abundance in the PVN of 6-OHDA rats compared with that of sham rats. In the PVN, a decrease in c-Fos expression was reported in unilateral PD model rats following formalin administration into the hind paw [6]. The PVN and supraoptic nuclei also produce oxytocin, a posterior pituitary hormone [17]. Oxytocin is reported to have an analgesic effect on nociceptive responses after intrathecal or systemic administration [18]. In addition, the oxytocin receptor has been shown to be involved in analgesia induced by oxytocin [19], and the oxytocin receptor is thought to be present in the spinal dorsal horn, dorsal root ganglia [20] and trigeminal root ganglia [21]. It was reported that the number of oxytocin-immunoreactive neurons in the PVN of patients with PD is lower than that in control subjects [22]. This decrease may be associated with a reduction in oxytocin production, and a decrease in its analgesic effect.

There are few studies investigating nociception using bilateral PD model rats [7–9]. Because idiopathic PD is bilateral, bilateral PD model rats are more closely to the pathological condition in humans [10]. Therefore, bilateral PD model rats might be more closely to patients with PD than unilateral PD model. However, we had a difficulty about producing bilateral PD model rats. It was difficult to accurately inject 6-OHDA bilaterally into the MFB. Although we injected 6-OHDA to 68 rats, which is similar number of rats used in our previous study, 10 rats showed bilateral dopaminergic denervation, and 54 rats showed unilateral dopaminergic denervation, and 4 rats died. Severe dopaminergic denervation may have occurred in the 4 dead rats. The mortality rate of bilateral 6-OHDA–injected rats was reported over 60% and, the number of TH-IR cells in the SNpc in 6-OHDA–injected rats significantly decreased to one–eighth (87.5% loss) compared with sham rats [13]. In the present study, we found 60% loss of TH-IR cells in the SNpc, the degree of TH-IR cell loss may be mild compared with the results of Bonato et al. [13]. When we used unilateral PD model rats in our previous study [3], we did not have this difficulty and no rats that received 6-OHDA injection died. This may be the reason why the studies using bilateral PD model rats are a few and the studies using unilateral PD model rats are large.

However, in the study using unilateral PD model rats, the laterality of nociception may be considered. Several studies reported the change of nociception ipsilateral or contralateral side to the 6-OHDA lesion [5, 6, 23]. Although the issue of laterality has been considered, it is controversial. In the study using bilateral PD model rats, this issue may be excluded, and this may be a merit of the study using bilateral PD model rats.

The change of nociception was reported in the studies using unilateral PD model rats [4–6]. And, mechanical allodynia or hypersensitivity were reported in the studies using bilateral PD model rats [7–9]. Our results are consistent of results of these studies in that the change of nociception occurred.

The basal ganglia are thought to be involved in processing of nociceptive stimuli [2]. Dopamine is suggested to have antinociceptive effect through the dopamine D2 receptors [24, 25], and the electrical and chemical stimulation of the striatum is thought to produce an analgesic action through the dopamine D2 receptors [26]. In addition, the dopamine D2 receptor agonists produce an antinociceptive effect, and the dopamine D2 receptor antagonists attenuate the antinociceptive effect induced by the dopamine D2 receptor agonists [27, 28]. The PD rat model developed by 6-OHDA injection showed a moderate increase in the dopamine D1 receptor density and significant increase in the dopamine D2/D3 receptor density in the striatum [29]. Nigrostriatal dopamine denervation may lead to changes in dopamine transmission via the dopamine D2 receptor, which may have caused hyperalgesia observed in the present study.

Interactions between GABA, glutamate and dopamine have been suggested to affect the basal ganglia [30, 31]. In addition to dopamine, other neurotransmitters may be involved in nonmotor symptoms, including the pain experienced with PD [32]. Both patients with PD and experimental models exhibit disturbances of the GABAergic system [33, 34], and the level of GABA in the SN was reported to increase in unilateral PD model rats [35]. Plasma levels of aspartate and glutamate were seen to be lower in patients with PD [36]. The administration of 6-OHDA may cause changes in the transmission of dopamine as well as GABA and glutamate, which may be involved in alteration of nociception.

In conclusion, when patients with PD receive nociceptive stimuli, an increase in the activity of the trigeminal spinal subnucleus caudalis may occur, leading to hyperalgesia. In addition, the results of our study suggested the PVN is also involved in the hyperalgesia process.

## Supporting information

S1 Fig Photomicrograph of striatum of sham rat. (TIF)

S2 Fig Photomicrograph of striatum of 6-OHDA rat. (TIF)

S3 Fig Photomicrograph of substantia nigra of sham rat. (TIF)

S4 Fig Photomicrograph of substantia nigra of 6-OHDA rat. (TIF)

S5 Fig Photomicrograph of substantia nigra pars compacta of sham rat. (TIF)

S6 Fig Photomicrograph of substantia nigra pars compacta of 6-OHDA rat. (TIF)

S7 Fig Photomicrograph of trigeminal spinal subnucleus caudalis of sham rat with non-stimulation. (TIF)

S8 Fig Photomicrograph of trigeminal spinal subnucleus caudalis of 6-OHDA rat with non-stimulation. (TIF)

S9 Fig Photomicrograph of trigeminal spinal subnucleus caudalis of sham rat with formalin administration. (TIF)

S10 Fig Photomicrograph of trigeminal spinal subnucleus caudalis of 6-OHDA rat with formalin administration. (TIF)

S11 Fig Photomicrograph of paraventricular nucleus of sham rat with non-stimulation. (TIF)

S12 Fig Photomicrograph of paraventricular nucleus of sham rat with formalin administration. (TIF)

S13 Fig Photomicrograph of paraventricular nucleus of 6-OHDA rat with non-stimulation. (TIF)

S14 Fig Photomicrograph of paraventricular nucleus of 6-OHDA rat with formalin administration. (TIF)

S1 File Raw data of behavioral and immunohistochemical responses. (XLSX)
