# Supplementary figures and images for: Bilateral Parkinson’s disease model rats exhibit hyperalgesia to subcutaneous formalin administration into the vibrissa pad
Source: PLoS One. 2019 Dec 5;14(12):e0225928. doi: 10.1371/journal.pone.0225928 (PMC6894844; doi:10.1371/journal.pone.0225928)

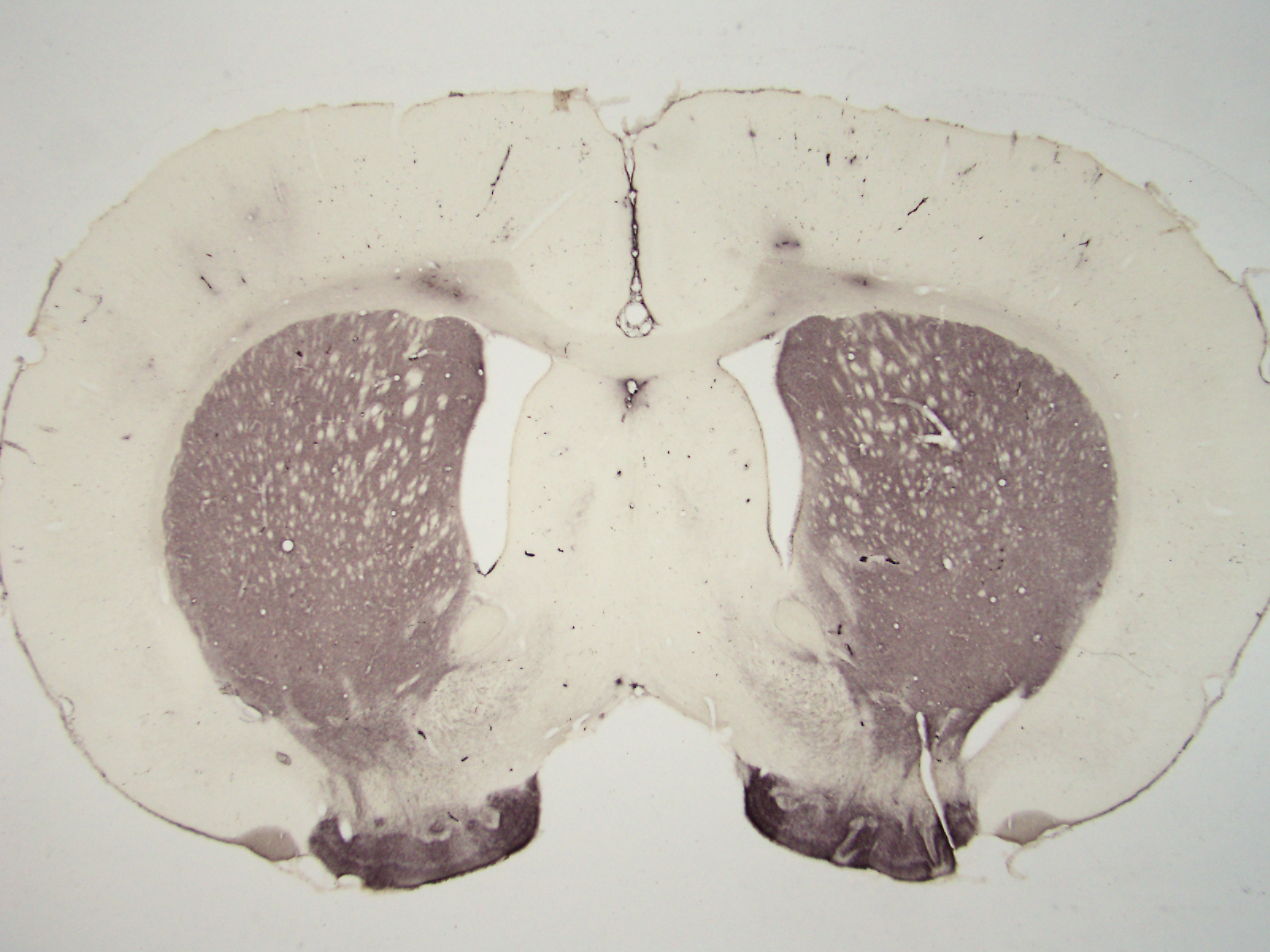

Supplement: S1 Fig — (TIF) [file pone.0225928.s001.TIF]

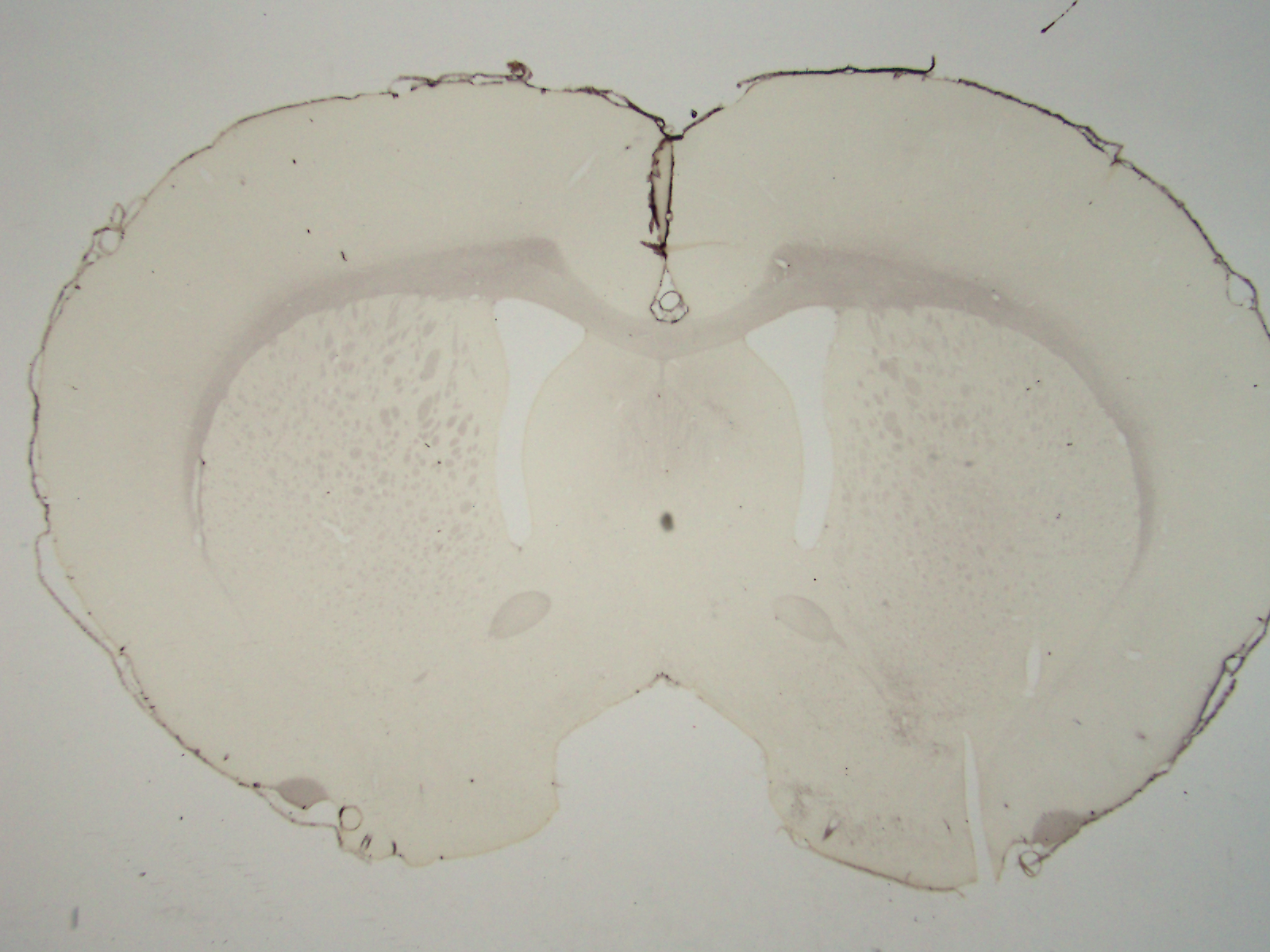

Supplement: S2 Fig — (TIF) [file pone.0225928.s002.TIF]

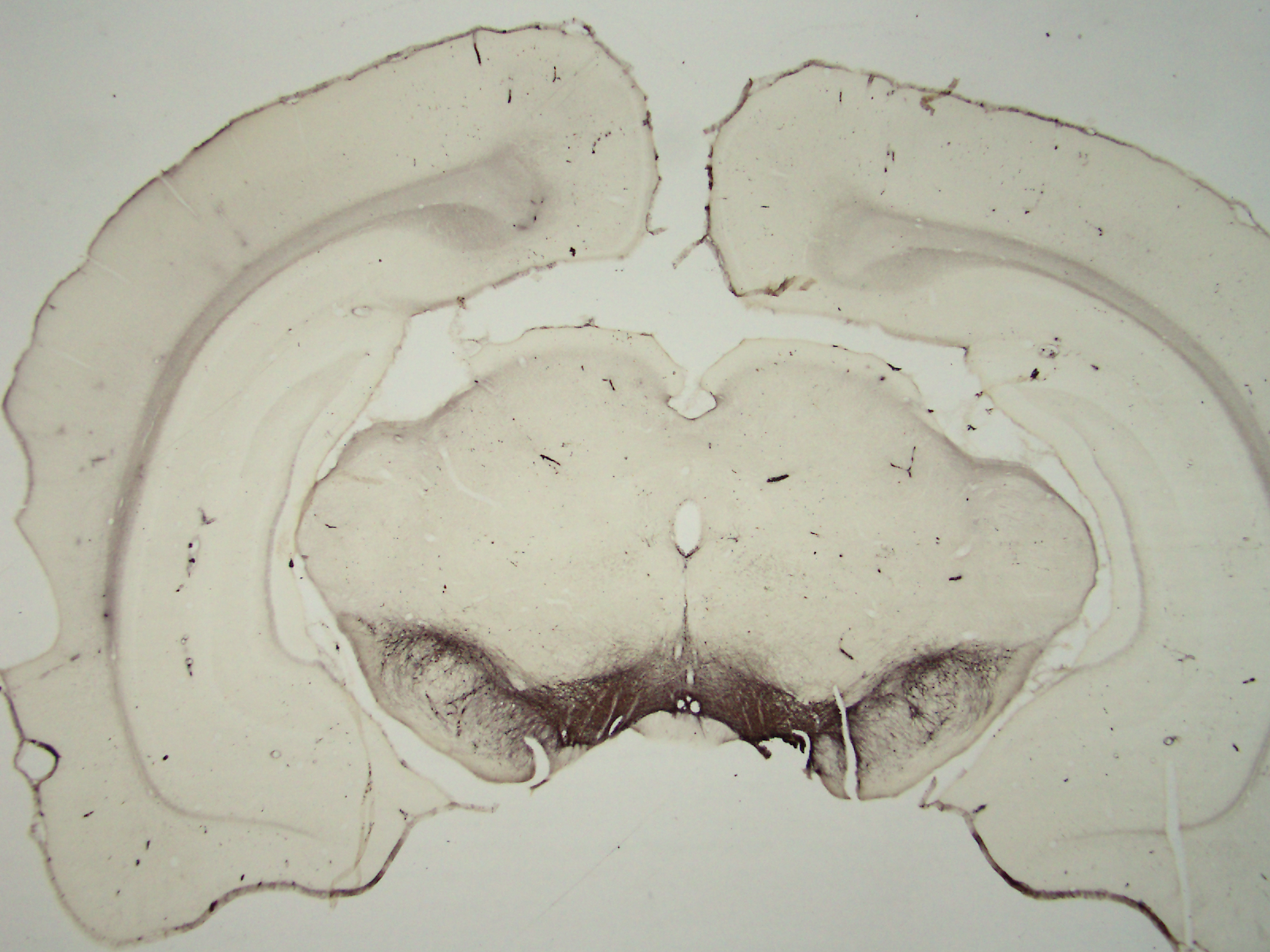

Supplement: S3 Fig — (TIF) [file pone.0225928.s003.TIF]

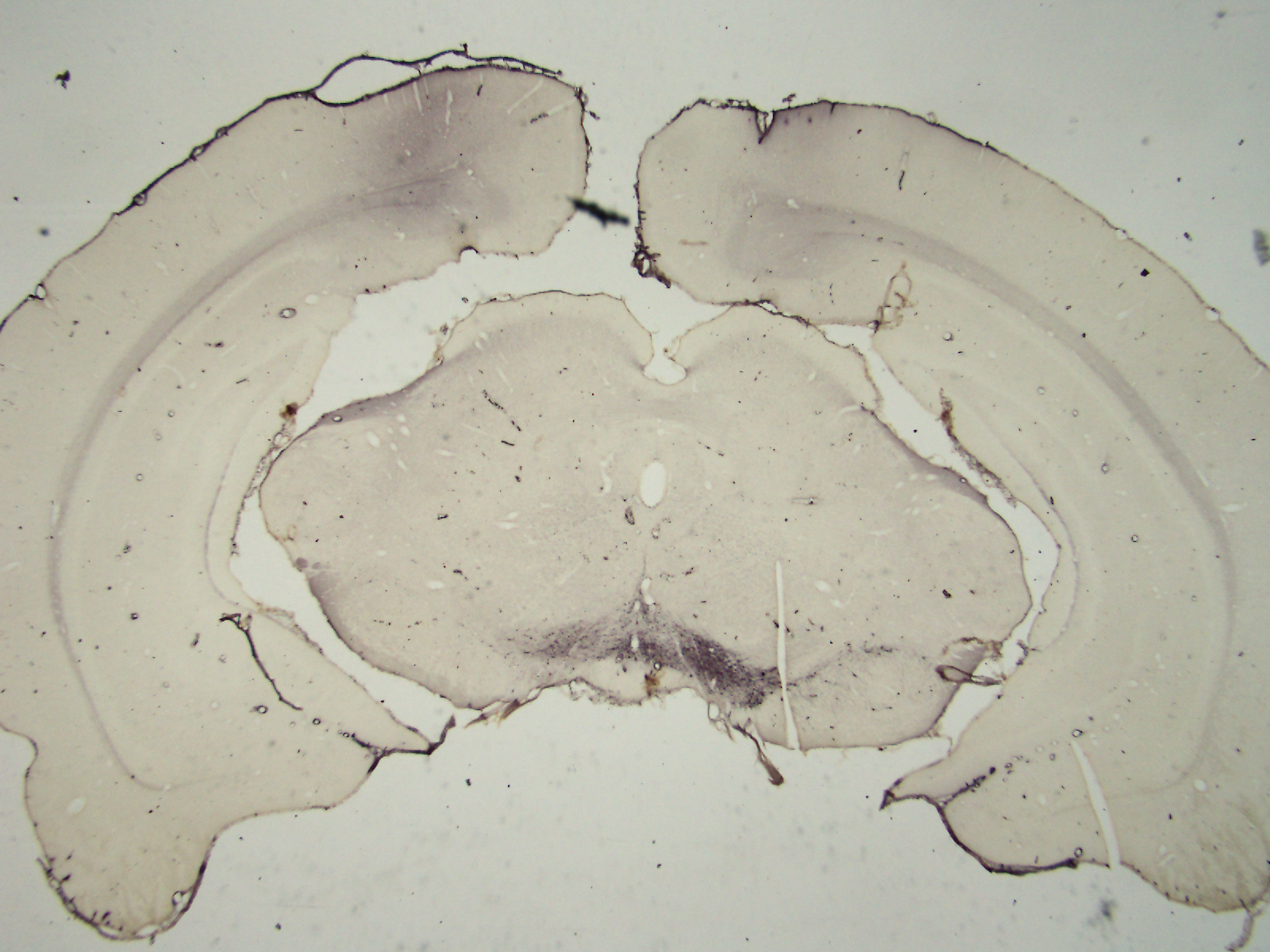

Supplement: S4 Fig — (TIF) [file pone.0225928.s004.TIF]

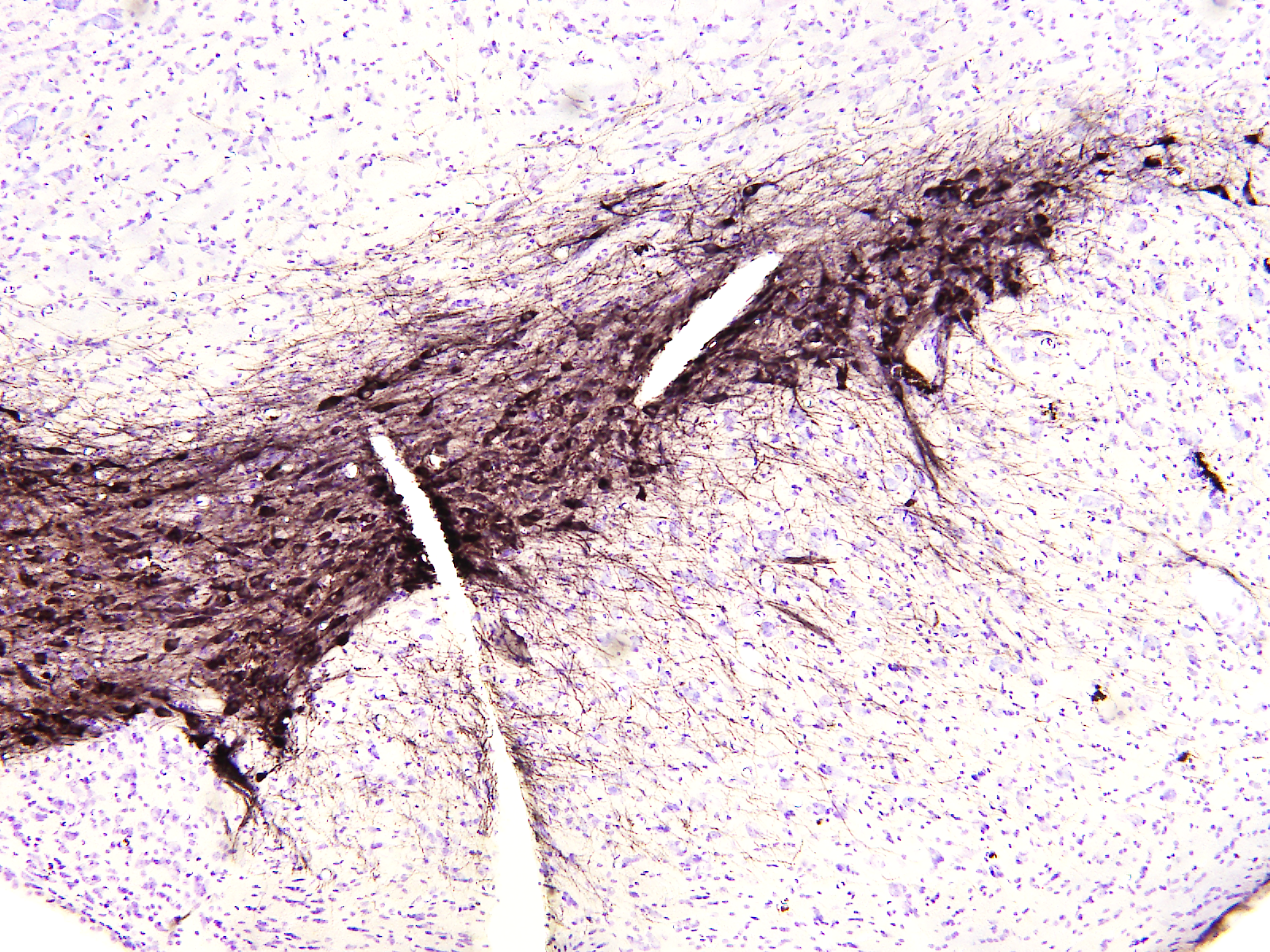

Supplement: S5 Fig — (TIF) [file pone.0225928.s005.TIF]

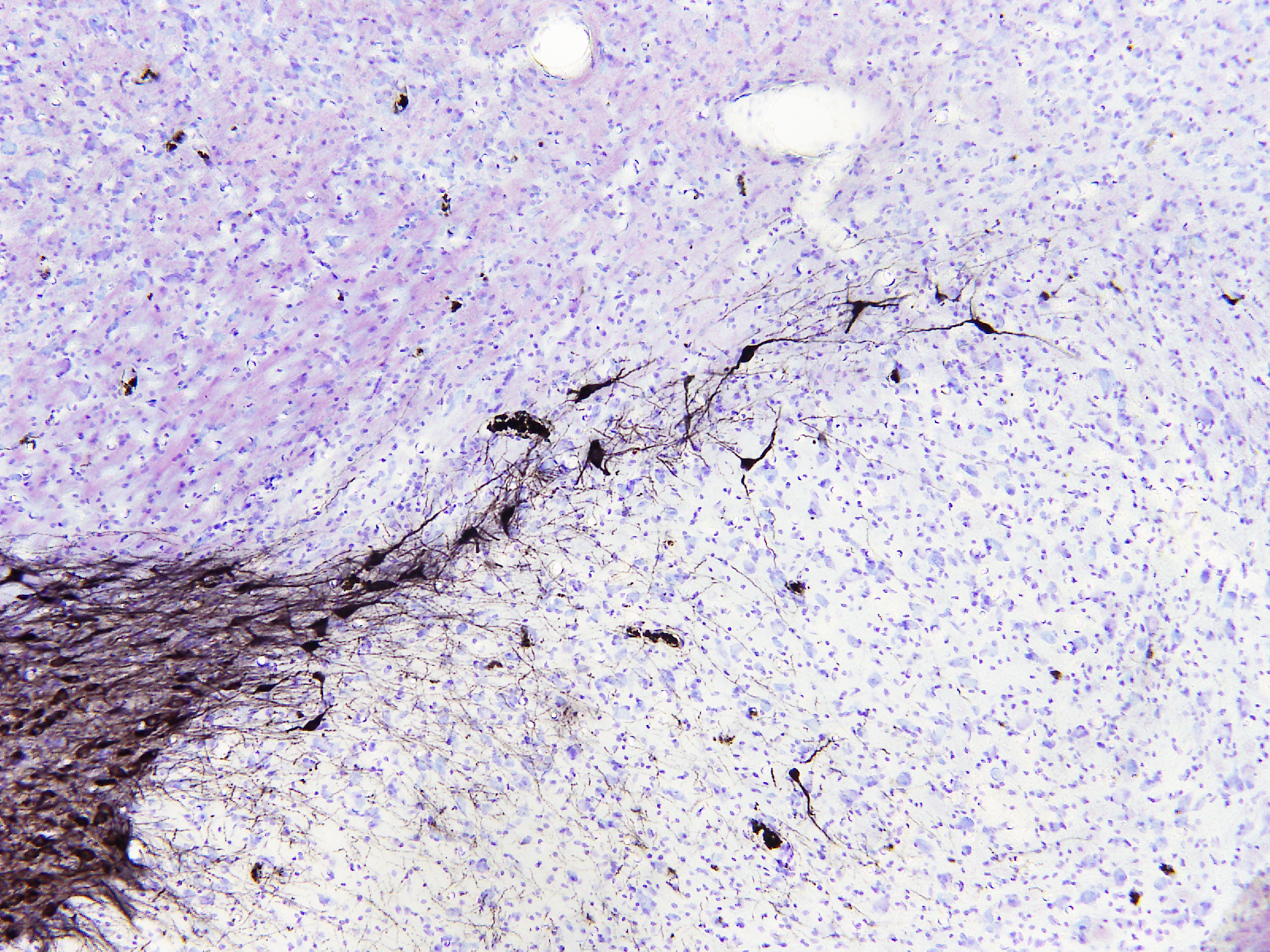

Supplement: S6 Fig — (TIF) [file pone.0225928.s006.TIF]

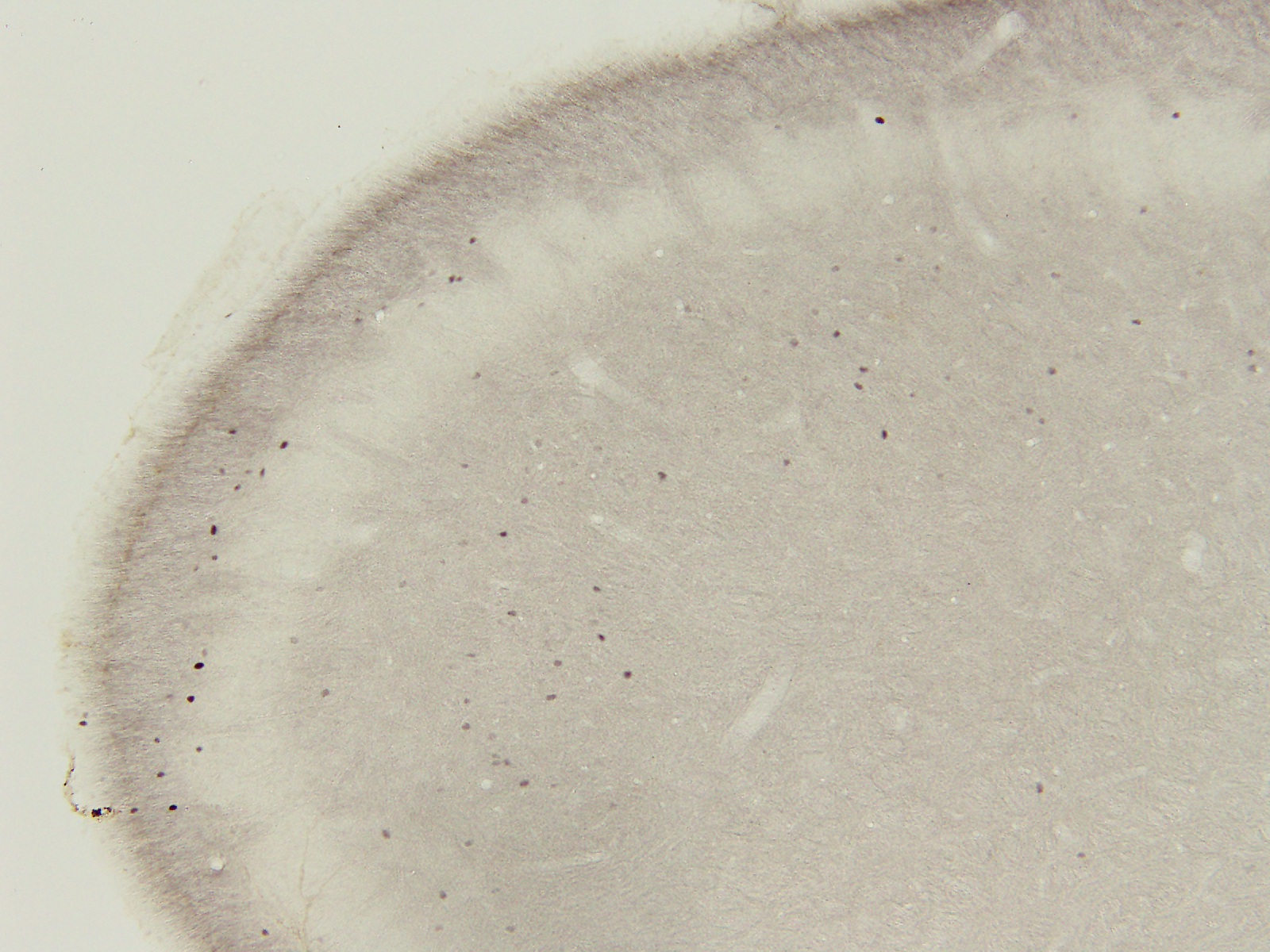

Supplement: S7 Fig — (TIF) [file pone.0225928.s007.TIF]

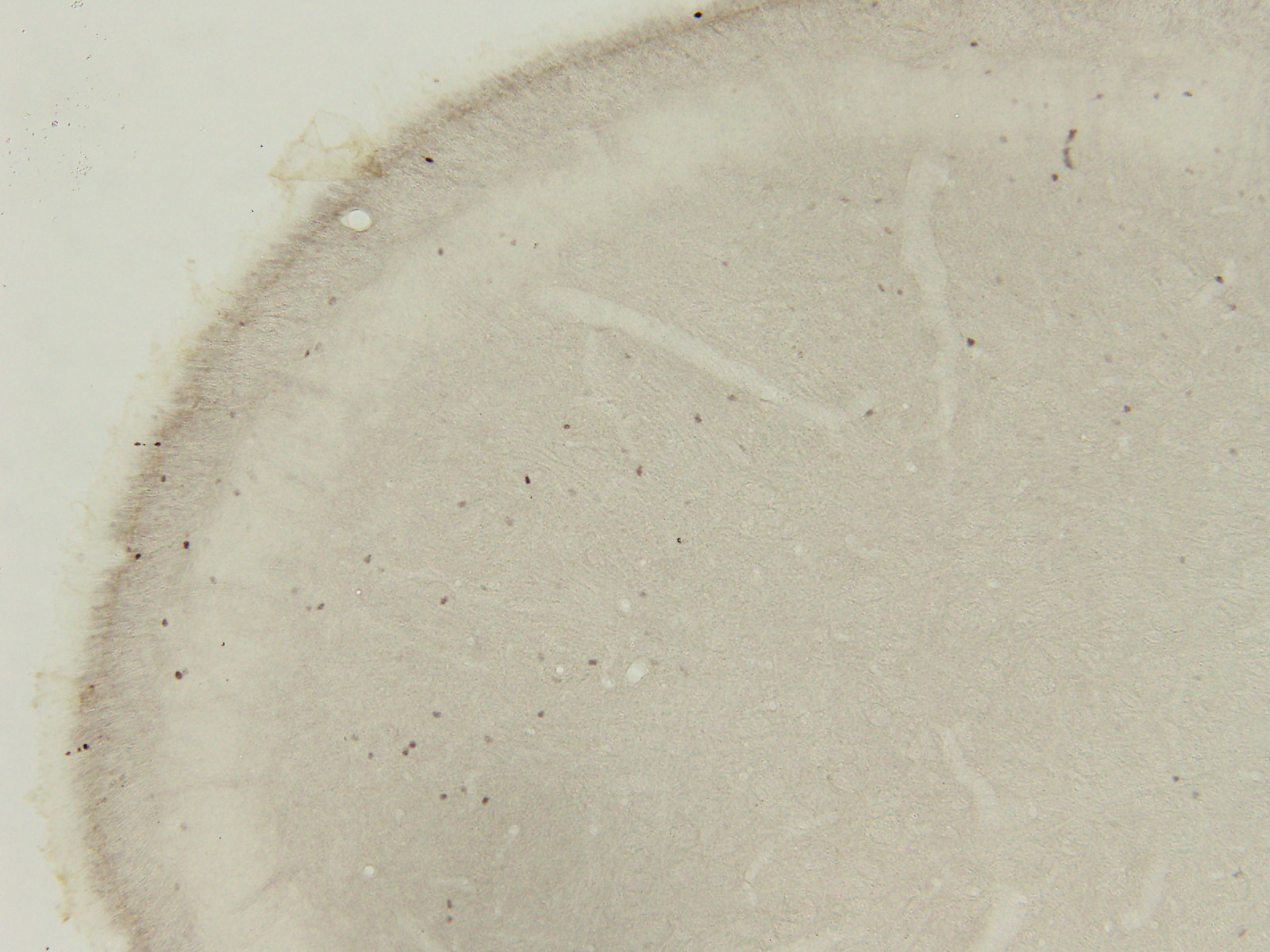

Supplement: S8 Fig — (TIF) [file pone.0225928.s008.TIF]

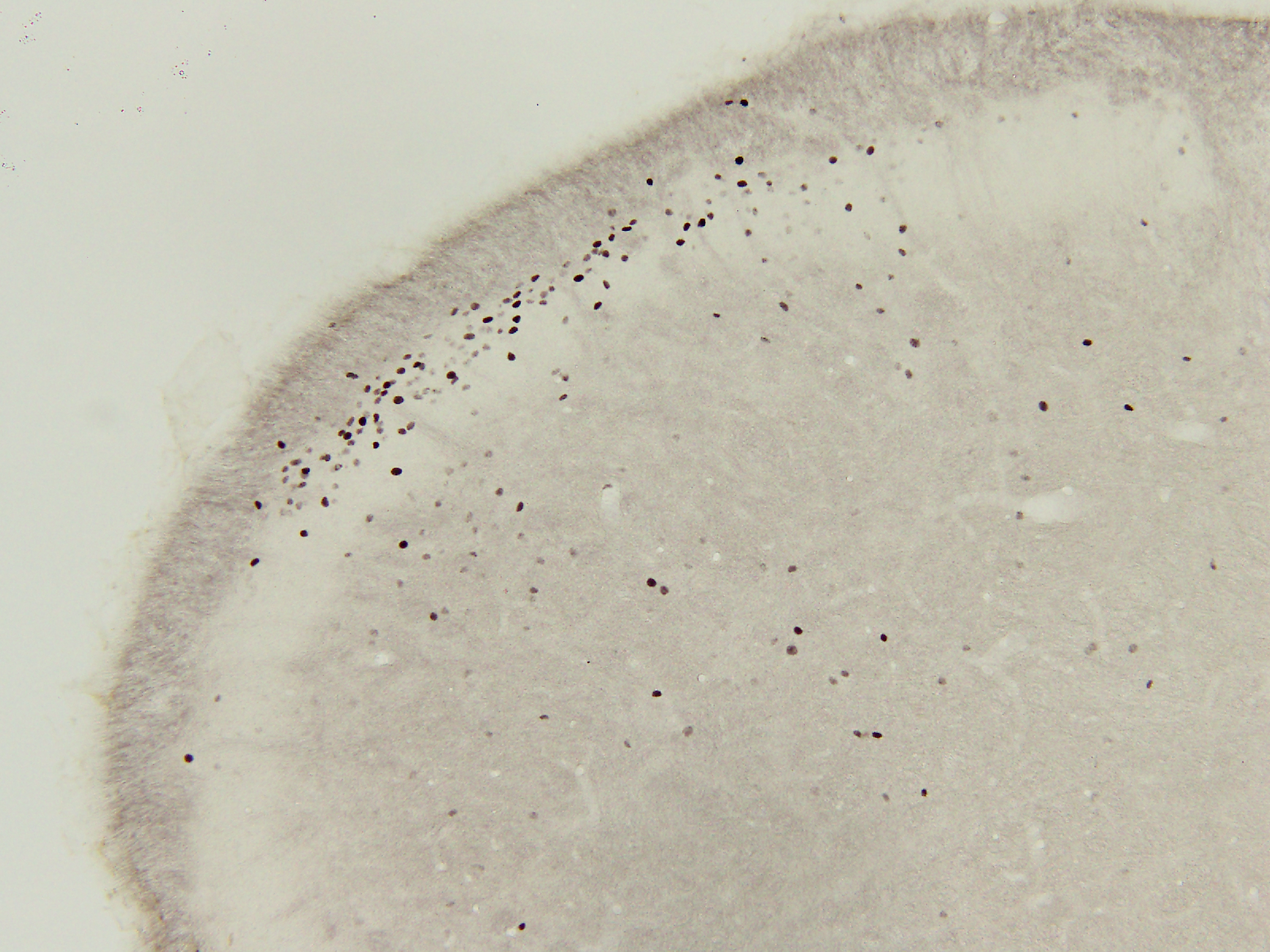

Supplement: S9 Fig — (TIF) [file pone.0225928.s009.tif]

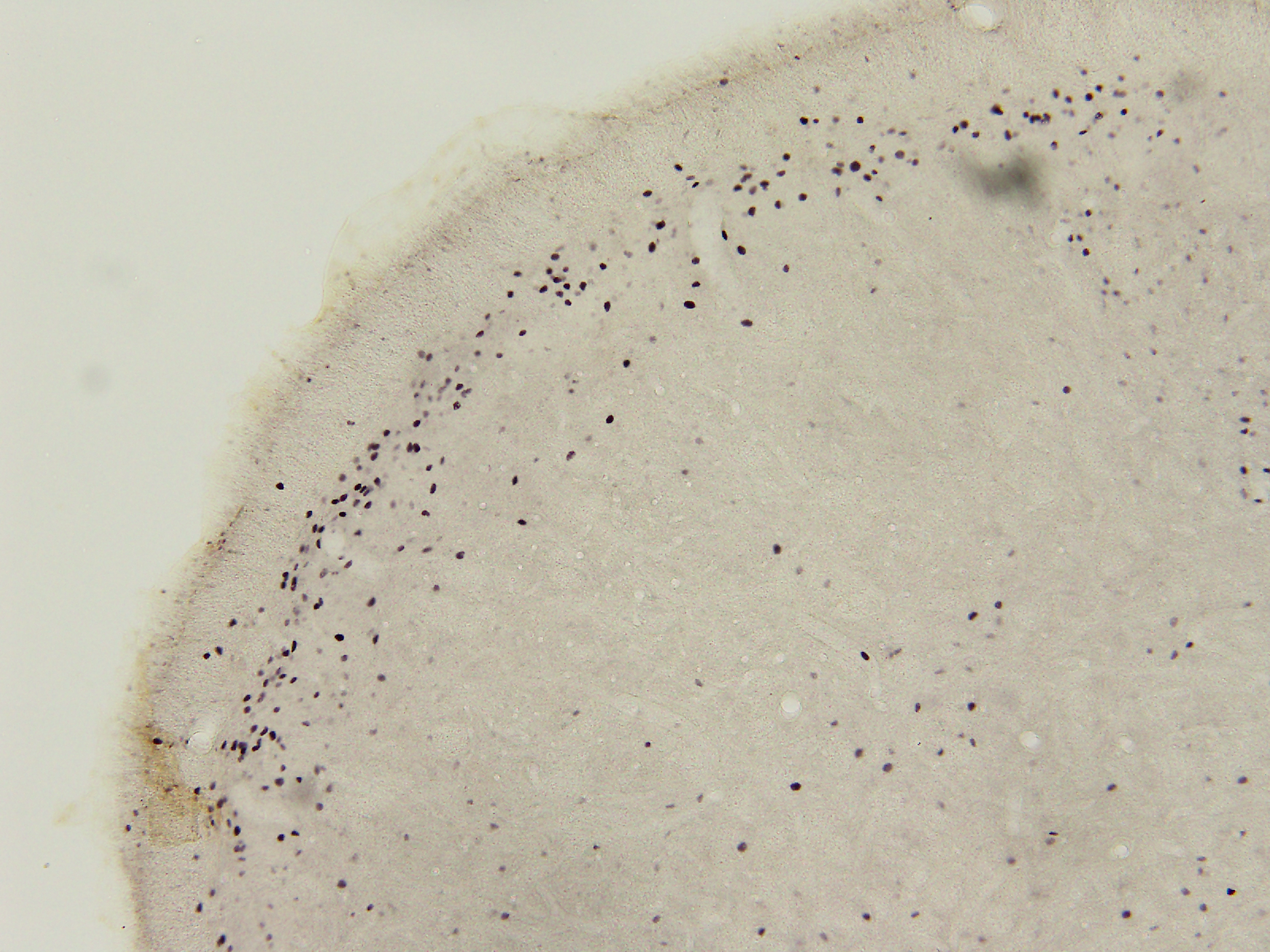

Supplement: S10 Fig — (TIF) [file pone.0225928.s010.TIF]

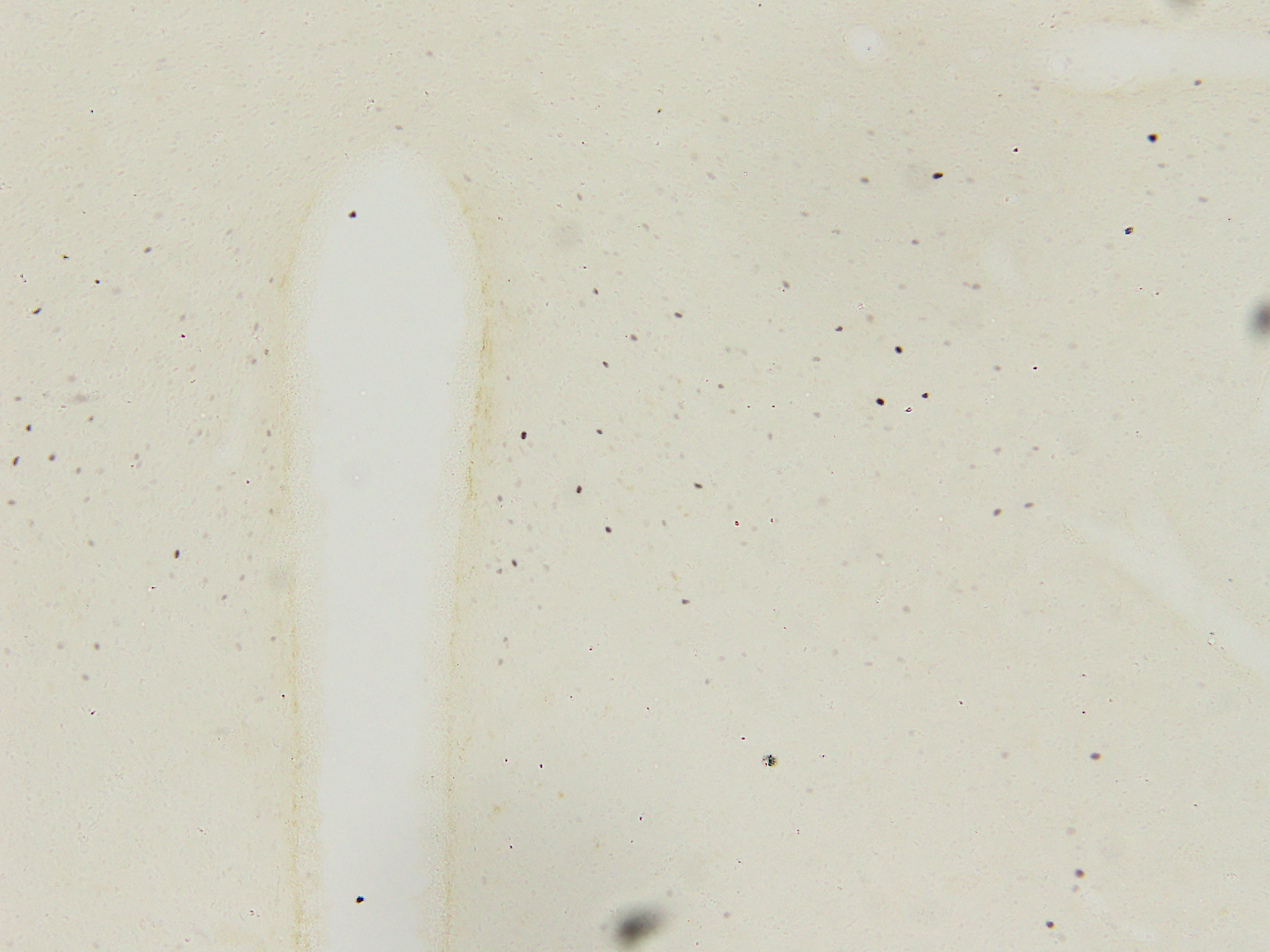

Supplement: S11 Fig — (TIF) [file pone.0225928.s011.TIF]

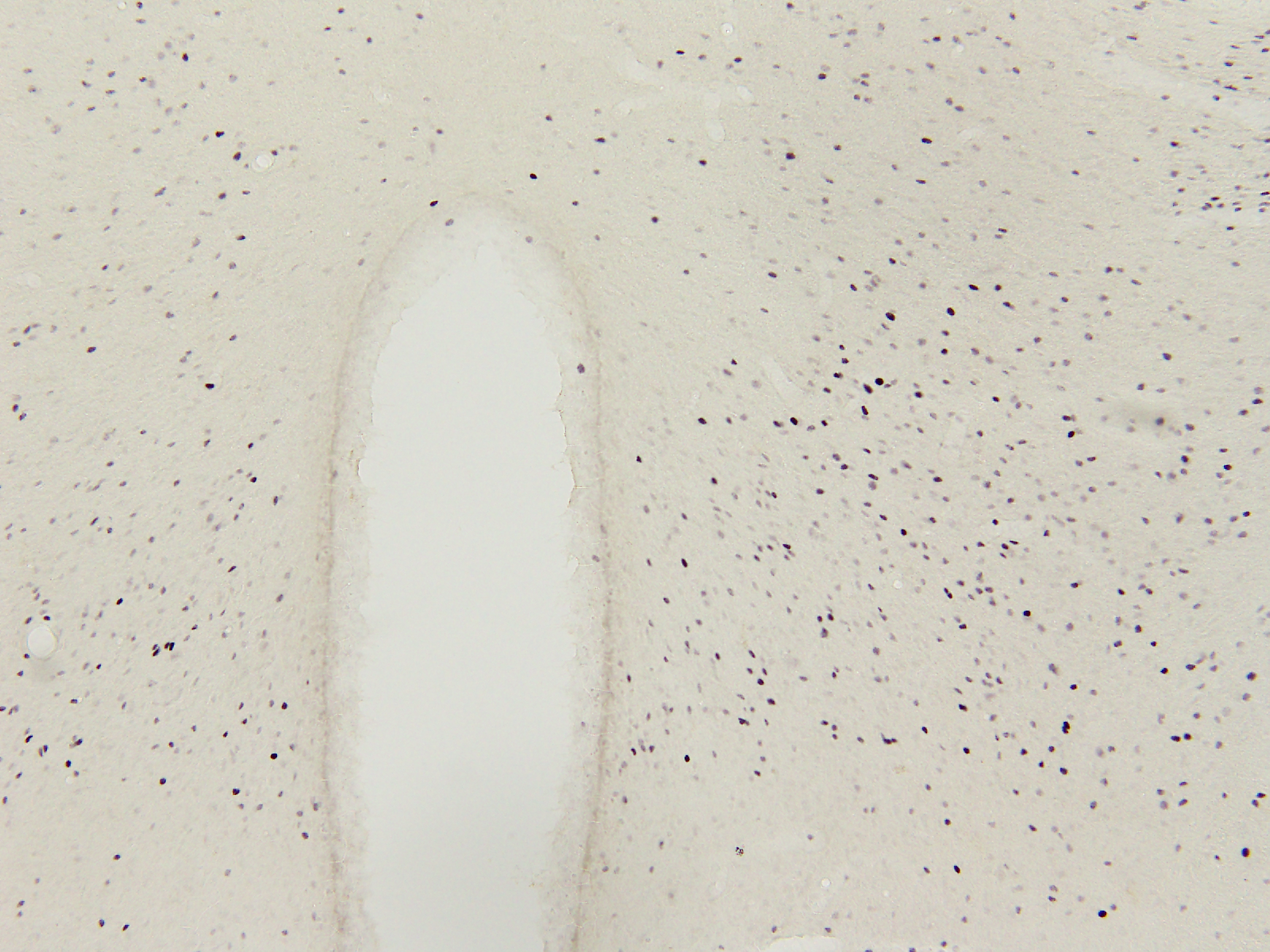

Supplement: S12 Fig — (TIF) [file pone.0225928.s012.TIF]

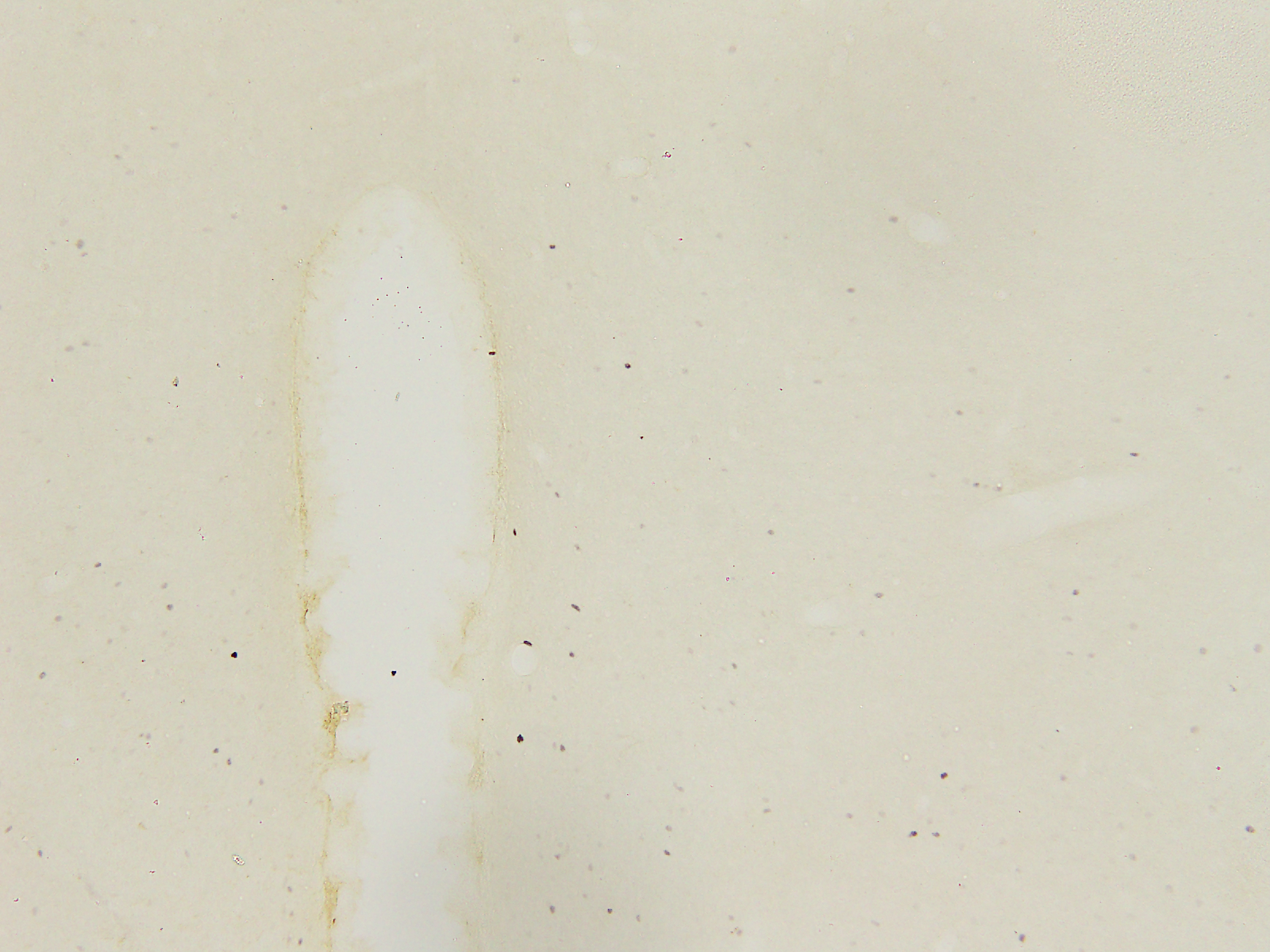

Supplement: S13 Fig — (TIF) [file pone.0225928.s013.TIF]

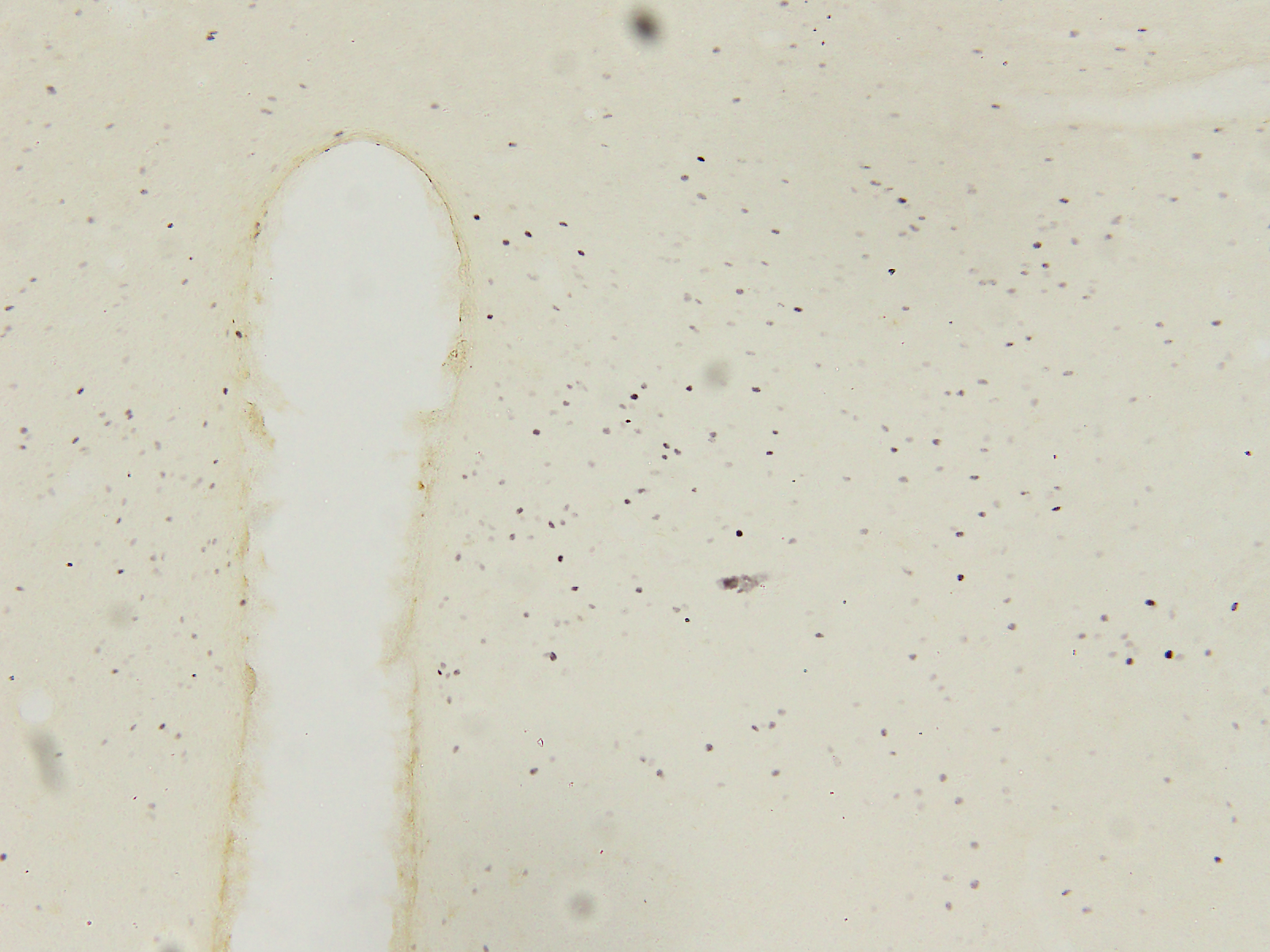

Supplement: S14 Fig — (TIF) [file pone.0225928.s014.TIF]
